# Effect of Acute Cold Stress on Neuroethology in Mice and Establishment of Its Model

**DOI:** 10.3390/ani12192671

**Published:** 2022-10-04

**Authors:** Yajie Hu, Yang Liu, Shize Li

**Affiliations:** National Experimental Teaching Demonstration Center of Animal Medicine Foundation, College of Animal Science and Veterinary Medicine, Heilongjiang Bayi Agricultural University, Daqing 163319, China

**Keywords:** acute cold stress, neuroethology, open field test, plus maze test, hormone

## Abstract

**Simple Summary:**

Unavoidable cold stress has widespread and complex effects on humans and animals in cold regions. A series of abnormal changes in behavior, emotion and neuroendocrine system occur in response to cold stress. However, the neglect of these physiological changes and the difficulty of defining cold stress combine to hinder the in-depth study and understanding of neurobehavior under cold stress. Therefore, our study established cold-stress models of mice with different intensities to systematically observe the neurobehavioral changes and to summarize the neurobehavioral characteristics and patterns. The results of the open field test and elevated plus maze test show that mild acute cold exposure promoted spontaneous movements, increased exploratory behaviors, and improved anxiety. As the intensity of cold exposure increased, cold exposure negatively affected spontaneous movements, exploratory behaviors and anxiety emotion. Combined with the relevant stress hormones, the activation of the hypothalamic–pituitary–adrenal axis and locus coeruleus-noradrenergic system with varying degrees were found to underlie these behavioral and emotional fluctuations. This study provides new insights into the interaction pattern between animals and the environment, and the understanding is beneficial to promoting animal welfare and its assessment in cold regions.

**Abstract:**

Cold environment is an inevitable stress source for humans and livestock in cold areas, which easily induce a cold stress response and then cause a series of abnormal changes in energy metabolism, neuroendocrine system, behavior and emotion. Homeostasis is maintained by the unified regulation of the autonomic nervous system, endocrine system, metabolism and behavior under cold exposure. Behavior is an indispensable part of the functional regulation of the body to respond to environmental changes. At present, the behavioral changes caused by cold exposure are unclear or even chaotic due to the difficulty of defining cold stress. Therefore, this study aims to systematically observe the changes in spontaneous movement, exploratory behavior and anxiety of mice under different intensity cold exposure and summarize the characteristics and behavior traits combined with relevant blood physiological indexes under corresponding conditions. Mice models of cold stress with different intensities were established (cold exposure gradients were 22 °C, 16 °C, 10 °C and 4 °C, and time gradients of each temperature were 2 h, 4 h, 6 h, 8 h, 10 h and 12 h). After the corresponding cold exposure treatment, mice immediately carried out the open field test(OFT) and elevated plus maze test (PMT) to evaluate their spontaneous movement, exploratory behavior and anxiety. Subsequently, blood samples were collected and used for the determination of corticosterone (Cort), corticotropin-releasing hormone (CRH), epinephrine (E), norepinephrine (NE), dopamine (DA) and 5-hydroxytryptamine (5-HT) by enzyme-linked immunosorbent assay (ELISA). Spontaneous movement of mice increased under 22 °C cold exposure, but their exploration behavior did not significantly change, and their anxiety improved at the initial stage. The spontaneous movement and anxiety of mice increased in the initial stage and decreased in the later stage under cold exposure at 16, 10 and 4 °C and the exploratory behavior was inhibited. The hypothalamic–pituitary–adrenal (HPA) axis and locus coeruleus-noradrenergic (LC/NE) system were activated by cold stress and fluctuated with different intensities of cold exposure. Meanwhile, serum DA increased, and 5-HT was the opposite under different intensities of cold exposure. In conclusion, mild acute cold exposure promoted the spontaneous movement, increased exploratory behavior and improved anxiety. As the intensity of cold exposure increases, cold exposure had a negative effect on spontaneous movement, exploratory behavior and emotion. The physiological basis of these behavioral and emotional changes in mice under different intensity cold stimulation is the fluctuation of Cort, CRH, E, NE, DA and 5-HT.

## 1. Introduction

Any organism has its own unique environment temperature suitable for survival, and the organism can resist the change of environmental temperature in a certain range. Organisms are prone to stress under adverse environmental temperatures, for example, humans and animals in cold areas are susceptible to cold stress [1]. Cold stress induced by low temperature has a complex and wide effect, which causes a series of abnormal changes in energy metabolism [2,3], neuroendocrine system [4], immunity [5], antioxidant [6], behavior [7] and emotion. Behavior is also an important component for the body to adapt to environmental changes, which contribute to the stability of the internal environment under stress with neuroendocrinology and metabolism [8,9]. The central nervous system is the hub of regulating a cold stress response, such as energy homeostasis, body temperature maintenance and cold-induced thermogenesis [10,11]. The locus coeruleus-noradrenergic (LC/NE) system and hypothalamic–pituitary–adrenal (HPA) axis regulate a series of physiological processes in response to cold stress through a variety of classical stress hormones [12,13]. Studies have found that stress hormones such as corticosterone (Cort), corticotropin-releasing hormone (CRH), epinephrine (E) and norepinephrine (NE) in blood fluctuated under cold stress, and then they caused behavioral changes [14,15,16,17]. Dopamine (DA), 5-hydroxytryptamine (5-HT) and other neurotransmitters are also involved in regulating a variety of behaviors in this process, including fighting, ingestion and reproduction [18,19]. Previous studies have only focused on cold stress and animal maintenance behavior and presented it in a plainly descriptive manner. For example, cold-exposed turkeys spend more time curled up, shivering, preening, and bristling [20]. The feeding behavior of mice or pigeons increased during cold exposure [21,22]. However, prenatal cold stress leads to anxiety-like behavior changes in offspring [23,24]. This may be because prenatal cold stress inhibited the growth and development of hippocampal neurons in weaned rats, and thus, induced the reduction in anxiety in rats [25]. Similarly, cold stress causes persistent movement and behavioral deficits in Drosophila, which depend on the dose of cold exposure and persist for a long time after the stress subsides [26]. The initial response to cold exposure is a rapid drop in peripheral temperature and a strong constriction of blood vessels locally in the extremities [27]. This consideration led us to investigate the neurobehavioral effects of varying degrees of acute cold exposure on mice. The effects of temperature and duration of cold stress on spontaneous movement, exploratory behavior and anxiety in mice were evaluated, and the possible causes of these behaviors were analyzed in conjunction with serology. Observing stress-related anxiety in rodents often relies on species-specific behaviors such as increasing risk assessment, reducing exploration, seeking shelter, running away, burying or defecating. The open field test (OFT) reflects spontaneous movement and exploration behavior in this study. Plus maze test (PMT) assessed the emotion of anxiety.

## 2. Materials and Methods

### 2.1. Animals and Treatment

#### 2.1.1. Animal Management

Male specific pathogen-free institute of cancer research (ICR) mice (seven weeks old, 30–32 g) were purchased from the Experimental Animal Center of PLA Academy of Military Medical Sciences (Shenyang, China). The ICR mice were reared in the artificial intelligence climate room of animal science and Technology College of Heilongjiang Bayi Agricultural University for 7 days to adapt to the new environment. During the adaptive period, the mice were stroked for 15 minutes every day to avoid the extra stress, strangeness and other unrelated factors caused by the operation of the follow-up experiment. The ambient temperature is set at 28 ± 0.5 °C and the humidity is 40 ± 5%. Feed and water were provided ad libitum. The air exchange rate is 10–20 times/hour to keep the indoor air fresh. ICR mice were kept in polystyrene standard cages [cage size 375 mm (length) × 173 (width) mm × 165 (height)]. Each cage was given 200 g of soft sawdust bedding, the bedding material was changed twice a week and the cage and drinking water bottle were cleaned. Fluorescent lamp illumination, light intensity of 200 Lx, light dark cycle ratio of 12 h (light):12 h (dark) (light on 8:00 a.m., light off 8:00 p.m.). After the completion of adaptive feeding, the health status of ICR mice was observed and the mice with similar body conditions were screened. 

#### 2.1.2. Cold Exposure

**Figure 1:**
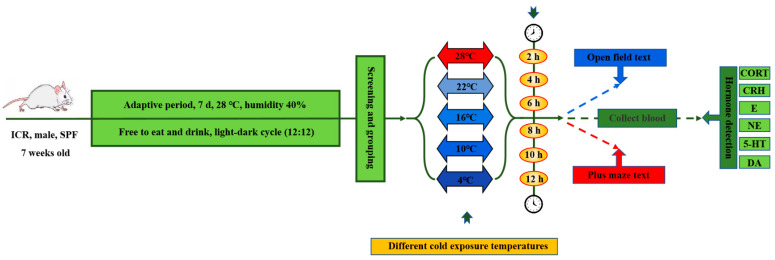
Study Protocol.

The temperature of acute cold exposure treatment was divided into four temperature gradients: 4 °C, 10 °C, 16 °C and 22 °C, which were compared with the 28 °C normal temperature control group. Each temperature gradient is subdivided into six time gradients: 2 h, 4 h, 6 h, 8 h, 10 h and 12 h. Each time gradient within each temperature gradient is a group consisting of 12 ICR mice. According to the group, the corresponding temperature and duration of cold exposure were carried out in the artificial climate chamber. After cold exposure, the behavior test was carried out immediately. The schematic diagram of the grouping and cold exposure procedure is shown in Figure 1.

### 2.2. Behavioral Measurement and Operating Procedures 

**Figure 2:**
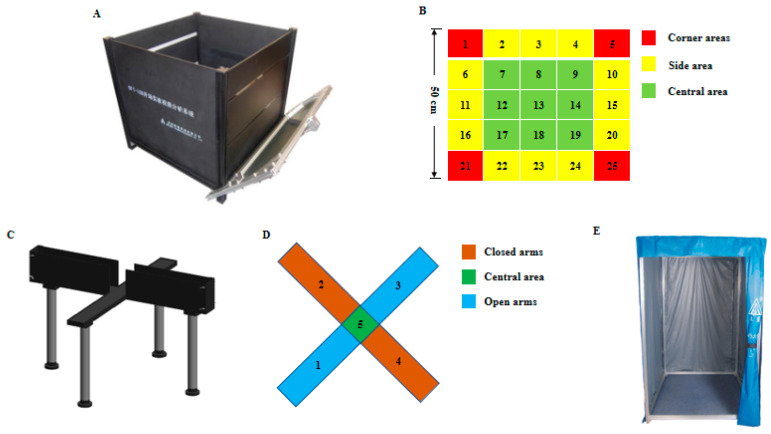
Behavioral test equipment. (**A**,**B**). Open field test device and its plane diagram. The external dimensions of the OFT box is 625 mm (length) × 740 mm (width) × 510 mm (height). The front and outer sides are equipped with reflectors. The bottom wall and surrounding side walls are made of black plastic; (**C**,**D**). Plus maze test device and its plane diagram. The elevated plus maze is composed of 2 open arms [300 mm (length) × 60 mm (width) × 6 mm (height)], 2 closed arms [300 mm (length) × 60 mm (width) × 150 mm (height)] and 1 central area [60 mm (length) × 60 mm (width)]. The height of the maze is 500 mm. Both the maze and the side walls of the closed arms are made of black plastic; (**E**). The floor type test station.

The TM vision behavioral test video analysis system, OFT box, elevated plus maze and floor type test station used in this test were purchased from Chengdu Taimeng Software Co., Ltd. Two 40 W fluorescent lamps are installed on both sides of the top of the box as the lighting system to maintain the same light conditions in the observation box; a high-definition camera connected with the TM vision behavior test video analysis system is fixed in the middle of the box top to collect and record images and test progress. The floor type test station is relatively independent of the external environment, which can reduce the impact of the environment, lighting, air flow, noise, human interference, etc., so all behavioral tests are conducted in the floor type test station. The schematic diagram of behavioral test equipment involved in this study is shown in Figure 2. 

In order to avoid the influence of the residual odor of the previous test mice on the next test, after each test, clean the feces, measure and clean the bottom and side wall of the test device, spray 75% alcohol and open ventilation at the same time, completely eliminate the odor left by the previous mouse until the test device is dry without odor. During the test, the operator’s operation should be stable and fast. If the operation is to find that the mouse starts to become irritable and restless, it is necessary to temporarily stop the action on the mouse and wait for it to calm down before starting the operation again. The strict control of environmental factors, such as the placement position of the mice was consistent. In order to avoid the influence of circadian rhythm on the test, all mice completed the corresponding test at the same time (8:00~11:00 a.m).

### 2.3. Blood Sample Collection and Determination of Serum Related Hormones

After the behavioral measurement of the corresponding cold exposure, the mice were euthanized to collect blood samples. Immediately, the serums were isolated and stored at –80 °C until analysis. The levels of serum Cort were determined by the commercial ELISA kit (#CEA540Ge 96T, Cloud-Clone Corp, Katy, TX, USA). The levels of serum CRH were determined by the commercial ELISA kit (#CEA835Mu 96T, Cloud-Clone Corp, Katy, TX, USA). The levels of serum E were determined by the commercial ELISA kit (#CEA858Ge 96T, Cloud-Clone Corp, Katy, TX, USA). The levels of serum NE were determined by the commercial ELISA kit (#CEA907Ge 96T, Cloud-Clone Corp, Katy, TX, USA). The levels of serum 5-HT were determined by the commercial ELISA kit (#CEA808G 96T, Cloud-Clone Corp, Katy, TX, USA). The levels of serum DA were determined by the commercial ELISA kit (#CEA851Ge 96T, Cloud-Clone Corp, Katy, TX, USA). All operations were carried out in strict accordance with the instructions of the kit.

### 2.4. Statistical Analysis

All data are expressed as the means ± standard error of the mean (SEM). Statistical analysis of the data was performed using GraphPad Prism software (La Jolla, CA, USA). Significant differences were evaluated by a one-way analysis of variance (ANOVA). For all analyses, post hoc comparisons were made using Fisher’s Least-Significant Difference (LSD) post hoc test. A *p* value < 0.05 was considered statistically significant.

## 3. Results

### 3.1. Changes in Spontaneous Movement of ICR Mice in Open Field Test under Different Intensity of Cold Exposure

**Figure 3:**
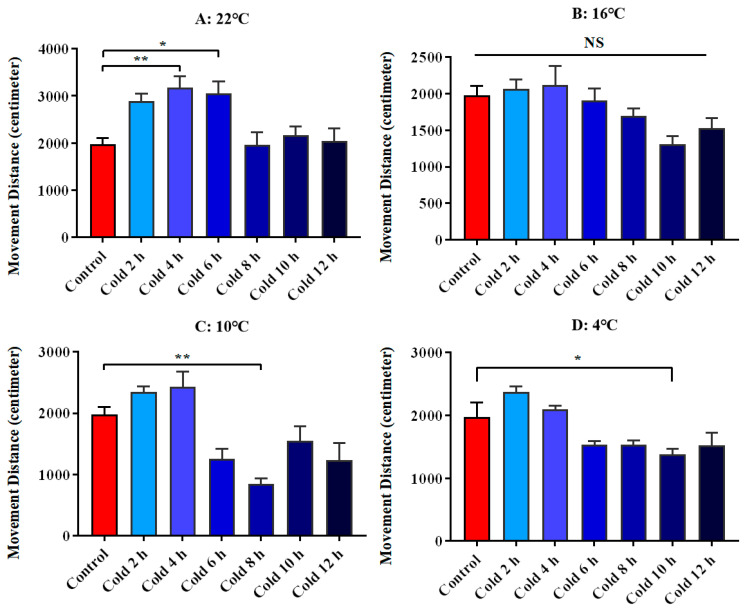
Changes of spontaneous movement of ICR mice under different intensity of cold exposure. (**A**). Changes in the movement distance of mice under cold exposure at 22 °C; (**B**). Changes in the movement distance of mice under cold exposure at 16 °C; (**C**). Changes in the movement distance of mice under cold exposure at 10 °C; (**D**). Change of movement distance of mice under cold exposure at 4 °C. Compared with 28 °C normal temperature control group, the difference was significant as * *p* < 0.05. ** *p* < 0.01.

We used the movement distance of ICR mice in the open field test as a parameter to evaluate their spontaneous movement. The results showed that spontaneous movement increased significantly at 22 °C for 4 h (*p* < 0.01) and 6 h (*p* < 0.05). There was no significant change at 16 °C. It was significantly decreased at 10 °C for 8 h (*p* < 0.01), and significantly decreased at 4 °C for 10 h (*p* < 0.05). All these results are shown in Figure 3.

### 3.2. Changes in Exploratory Behavior of ICR Mice in Open Field Test under Different Intensity of Cold Exposure 

**Figure 4:**
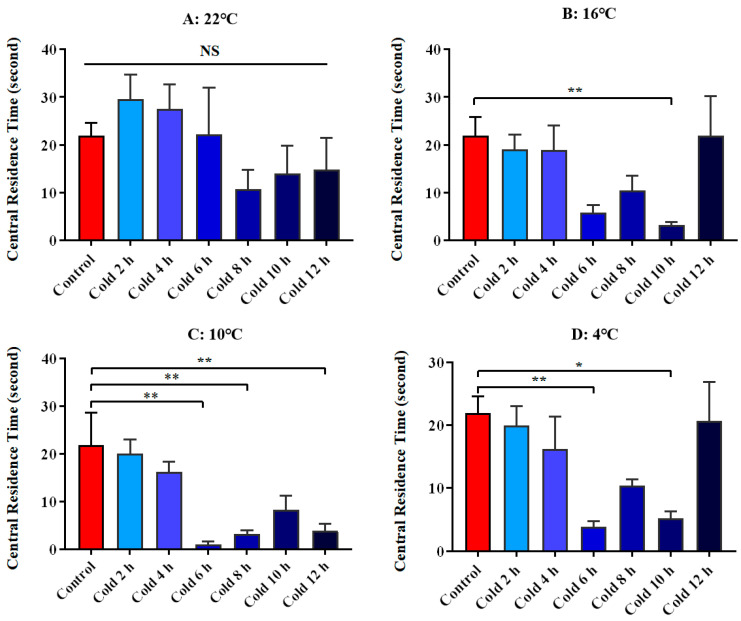
Changes in exploratory behavior of ICR mice under different intensity of cold exposure. (**A**). Changes in the central residence time of mice under cold exposure at 22 °C; (**B**). Changes in the central residence time of mice under cold exposure at 16 °C; (**C**). Changes in the central residence time of mice under cold exposure at 10 °C; (**D**). Change in the central residence time of mice under cold exposure at 4 °C. Compared with 28 °C normal temperature control group, the difference was significant as * *p* < 0.05. ** *p* < 0.01.

**Figure 5:**
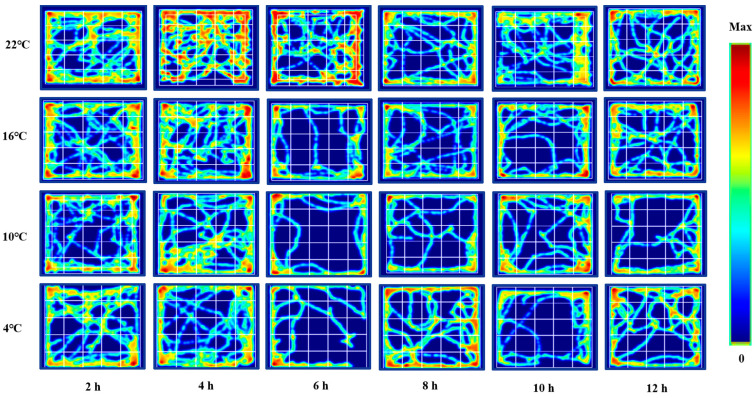
Movement trajectory map of mice in open field test under different intensity of cold exposure.

We used the central residence time of ICR mice in an open field test as a parameter to evaluate their exploratory behavior. There was no significant change at 22 °C. But it was significantly decreased at 16 °C for 10 h (*p* < 0.01). It was significantly reduced at 10 °C for 6 h, 8 h and 12 h (*p* < 0.01). It was significantly reduced at 4 °C for 6 h (*p* < 0.01) and 10 h (*p* < 0.05). All these results are shown in Figure 4. Meanwhile, Figure 5 shows the movement trajectories of mice in the open field test under different temperatures and times of cold exposure are shown, in order to more intuitively show the movement distance and regional distribution of mice in the open field experiment. Due to the length of the reasons not all listed, but the listed pictures have a good universality and representation.

### 3.3. Changes in Anxious Mood of ICR Mice in Elevated Plus Maze Test under Different Intensity of Cold Exposure

**Figure 6:**
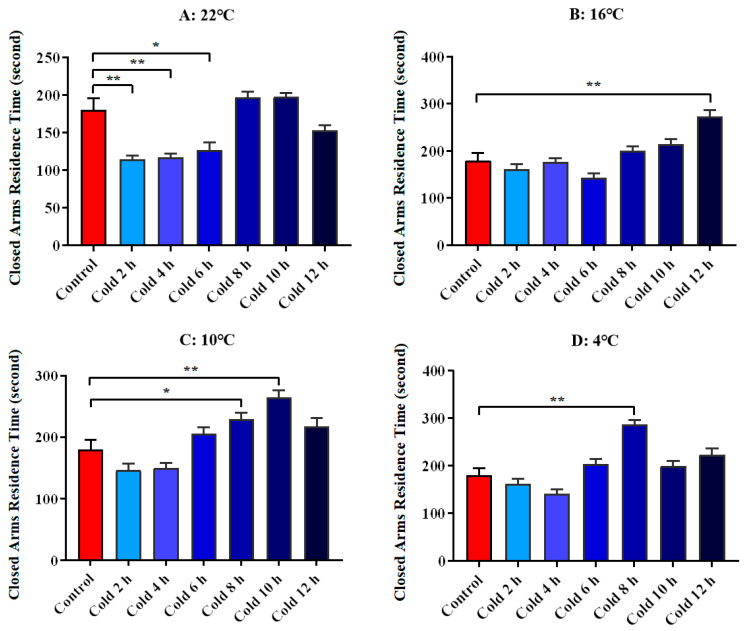
Changes in anxious mood of ICR mice under different intensity of cold exposure. (**A**). Changes in the closed arms residence time of mice under cold exposure at 22 °C; (**B**). Changes in the closed arms residence time of mice under cold exposure at 16 °C; (**C**). Changes in the closed arms residence time of mice under cold exposure at 10 °C; (**D**). Change in the closed arms residence time of mice under cold exposure at 4 °C. Compared with 28 °C normal temperature control group, the difference was significant as * *p* < 0.05. ** *p* < 0.01.

**Figure 7:**
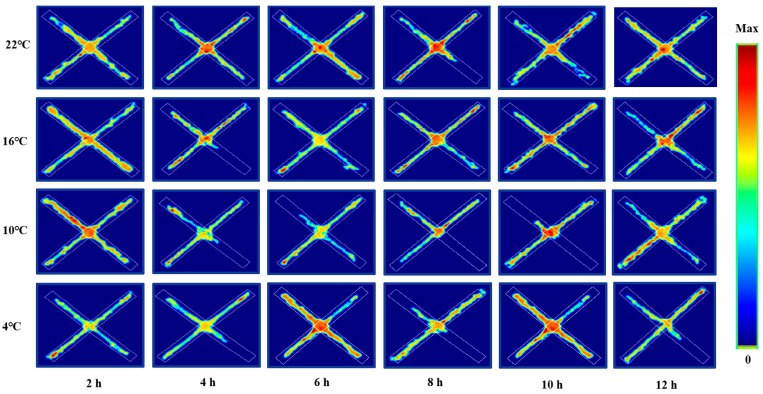
Movement trajectory map of mice in elevated plus maze test under different intensity of cold exposure.

We used the closed arm residence time of ICR mice in the elevated plus maze as a parameter to evaluate their anxiety level. The results showed that anxiety was significantly decreased at 22 °C for 2 h (*p* < 0.01), 4 h (*p* < 0.01) and 6 h (*p* < 0.05), significantly increased at 16 °C for 12 h (*p* < 0.01), increased significantly at 10 °C for 8 h (*p* < 0.05) and 10 h (*p* < 0.01), and increased significantly at 4 °C for 8 h (*p* < 0.01). All these results are shown in Figure 6. Meanwhile, Figure 7 shows the movement trajectories of mice in the elevated plus maze test under different temperatures and times of cold exposure are shown.

### 3.4. Changes in Related Hormones of ICR Mice in Elevated Plus Maze Test under Different Intensity of Cold Exposure

#### 3.4.1. Changes in Serum Cort and CRH of ICR Mice in Elevated Plus Maze Test under Different Intensity of Cold Exposure

**Figure 8:**
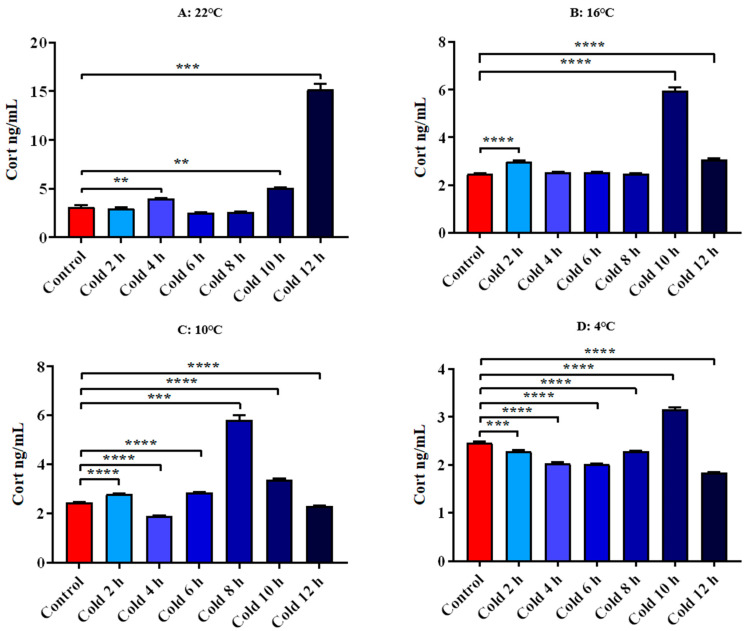
Changes in serum Cort of ICR mice under different intensity of cold exposure. (**A**). Changes in the serum Cort of mice under cold exposure at 22 °C; (**B**). Changes in serum Cort of mice under cold exposure at 16 °C; (**C**). Changes in the serum Cort of mice under cold exposure at 10 °C; (**D**). Change in the serum Cort of mice under cold exposure at 4 °C. Compared with 28 °C normal temperature control group, the difference was significant as ** *p* < 0.01. *** *p* < 0.001. **** *p* < 0.0001.

The results showed that Cort levels were significantly increased at 22 °C for 4 h, 10 h and 12 h (*p* < 0.01). After cold exposure at 16 °C for 2 h, 10 h and 12 h, it increased significantly (*p* < 0.0001). After cold exposure at 10 °C for 2 h, 6 h, 8 h, 10 h and 12 h, it increased significantly (*p* < 0.0001) and decreased significantly for 4 h (*p* < 0.0001). At 4 °C cold exposure for 2 h, 4 h, 6 h, 8 h and 12 h, they were significantly decreased (*p* < 0.001) and increased significantly for 10 h (*p* < 0.0001). All these results are shown in Figure 8.

**Figure 9:**
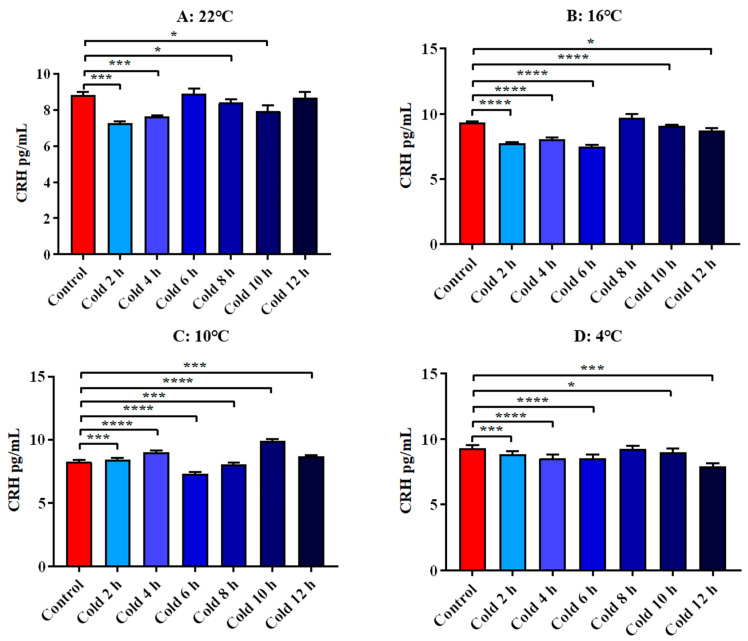
Changes in serum CRH of ICR mice under different intensity of cold exposure. (**A**). Changes in the serum CRH of mice under cold exposure at 22 °C; (**B**). Changes in serum CRH of mice under cold exposure at 16 °C; (**C**). Changes in the serum CRH of mice under cold exposure at 10 °C; (**D**). Change in the serum CRH of mice under cold exposure at 4 °C. Compared with 28 °C normal temperature control group, the difference was significant as * *p* < 0.05. *** *p* < 0.001. **** *p* < 0.0001.

The results showed that CRH levels were significantly decreased at 22 °C for 2 h, 4 h, 8 h and 10 h (*p* < 0.01). After cold exposure at 16 °C for 2 h, 4 h, 6 h 10 h and 12 h, it increased significantly (*p* < 0.01). After cold exposure at 10 °C for 2 h, 6 h, 8 h and 10 h, it decreased significantly (*p* < 0.01). At 4 °C cold exposure for 2 h, 4 h, 6 h, 10 h and 12 h, they were significantly decreased (*p* < 0.01). All these results are shown in Figure 9.

#### 3.4.2. Changes in Serum E and NE of ICR Mice in Elevated Plus Maze Test under Different Intensity of Cold Exposure

**Figure 10:**
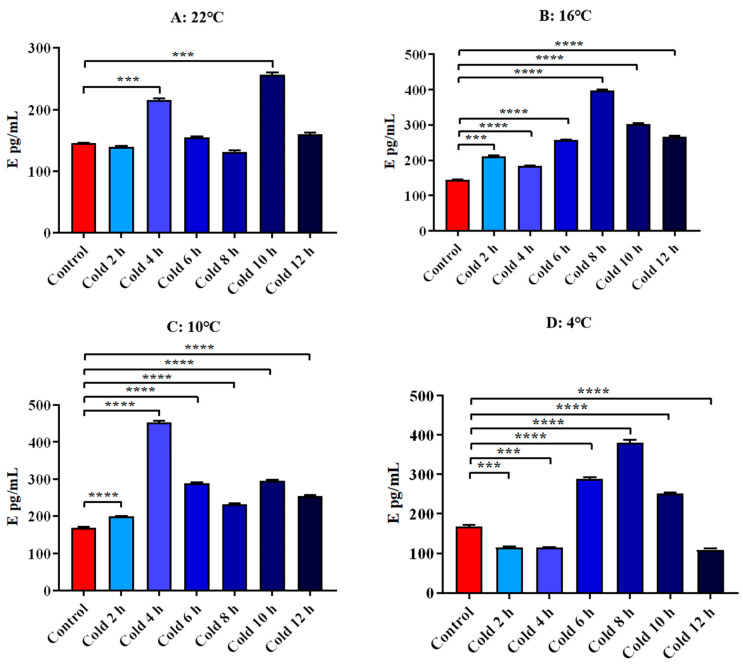
Changes in serum E of ICR mice under different intensity of cold exposure. (**A**). Changes in the serum E of mice under cold exposure at 22 °C; (**B**). Changes in serum E of mice under cold exposure at 16 °C; (**C**). Changes in the serum E of mice under cold exposure at 10 °C; (**D**). Change in the serum E of mice under cold exposure at 4 °C. Compared with 28 °C normal temperature control group, the difference was significant as *** *p* < 0.001. **** *p* < 0.0001.

The results showed that E levels were significantly increased at 22 °C for 4 h and 10 h (*p* < 0.001). After cold exposure at 16 °C, all time points were significantly increased (*p* < 0.001). After cold exposure at 10 °C, all time points were significantly increased. At 4 °C cold exposure for 6 h, 8 h and 10 h, it increased significantly (*p* < 0.0001). And it decreased significantly for 2 h, 4 h and 12 h (*p* < 0.001). All these results are shown in Figure 10.

**Figure 11:**
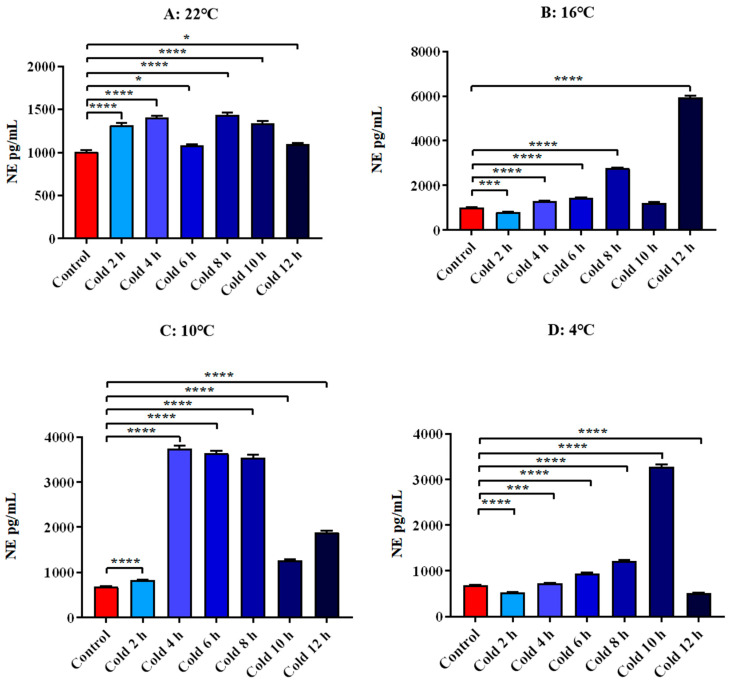
Changes in serum NE of ICR mice under different intensity of cold exposure. (**A**). Changes in the serum NE of mice under cold exposure at 22 °C; (**B**). Changes in serum NE of mice under cold exposure at 16 °C; (**C**). Changes in the serum NE of mice under cold exposure at 10 °C; (**D**). Change in the serum NE of mice under cold exposure at 4 °C. Compared with 28 °C normal temperature control group, the difference was significant as * *p* < 0.05. *** *p* < 0.001. **** *p* < 0.0001.

The level of NE in all time points was significantly increased at 22 °C. It was significantly increased at 4 h, 6 h, 8 h and 12 h after cold exposure at 16 °C (*p* < 0.0001). And it decreased significantly for 2 h (*p* < 0.001). After cold exposure at 10 °C, all time points were significantly increased (*p* < 0.0001). After 4 °C cold exposure for 4 h, 6 h, 8 h and 10 h, it increased significantly (*p* < 0.001). And it decreased significantly for 2 h and 12 h (*p* < 0.0001). All these results are shown in Figure 11.

#### 3.4.3. Changes in Serum 5-HT and DA of ICR Mice in Elevated Plus Maze Test under Different Intensity of Cold Exposure

**Figure 12:**
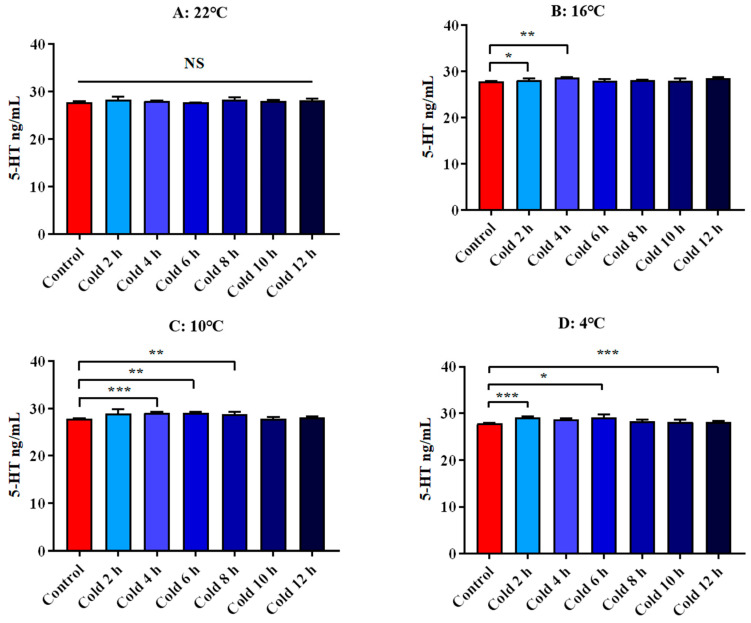
Changes of serum 5-HT of ICR mice under different intensity of cold exposure. (**A**). Changes in the serum 5-HT of mice under cold exposure at 22 °C; (**B**). Changes in serum 5-HT of mice under cold exposure at 16 °C; (**C**). Changes in the serum 5-HT of mice under cold exposure at 10 °C; (**D**). Change in the serum 5-HT of mice under cold exposure at 4 °C. Compared with 28 °C normal temperature control group, the difference was significant as * *p* < 0.05. ** *p* < 0.01. *** *p* < 0.001.

There was no significant difference in the level of 5-HT at each time point at 22 °C. The levels of 5-HT were significantly increased at 16 °C for 2 h and 4 h (*p* < 0.05). After cold exposure at 10 °C, it was significantly increased for 4 h, 6 h and 8 h. After cold exposure at 4 °C, it was significantly increased for 2 h, 6 h and 12 h. (*p* < 0.05). All these results are shown in Figure 12.

**Figure 13:**
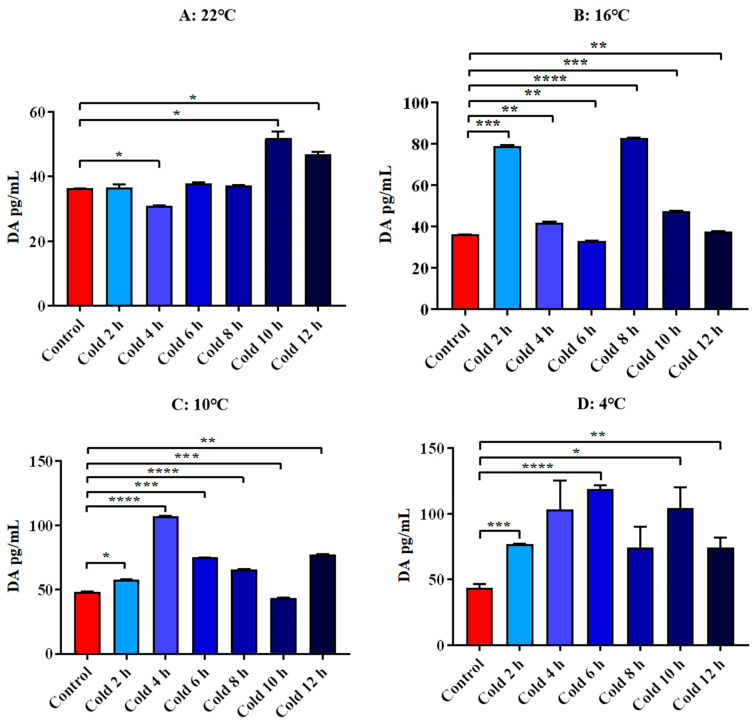
Changes of serum DA of ICR mice under different intensity of cold exposure. (**A**). Changes in the serum DA of mice under cold exposure at 22 °C; (**B**). Changes in serum DA of mice under cold exposure at 16 °C; (**C**). Changes in the serum DA of mice under cold exposure at 10 °C; (**D**). Change in the serum DA of mice under cold exposure at 4 °C. Compared with 28 °C normal temperature control group, the difference was significant as * *p* < 0.05. ** *p* < 0.01. *** *p* < 0.001. **** *p* < 0.0001.

The results showed that DA levels were significantly increased at 22 °C for 10 h and 12 h (*p* < 0.05). And it decreased significantly for 4 h (*p* < 0.05). After cold exposure at 16 °C, it was significantly increased for 2 h, 4 h, 8 h, 10 h and 12 h. And it decreased significantly for 6 h (*p* < 0.01). After cold exposure at 10 °C, it was significantly increased for 2 h, 4 h, 6 h, 8 h and 12 h. And it decreased significantly for 10 h (*p* < 0.001). After cold exposure at 4 °C, all time points were significantly increased. All these results are shown in Figure 13.

## 4. Discussion

OFT is one of the most commonly used platforms to measure behaviors in animal models. It is a fast and relatively easy test that provides a variety of behavioral information ranging from the general ambulatory ability to data regarding the emotionality of the subject animal [28,29]. The horizontal activity of mice reflects its spontaneous movement in the OFT of this study [30]. Our results showed that, initially, the mice were more active in locomotion to increase body temperature with a small decrease in ambient temperature, but as the ambient temperature decreased further, eventually the mice became hypothermic and the movement decreased significantly. Some studies suggest that a cold environment has certain disadvantages to the exercise intensity and endurance of animals. Unpredictable chronic stress reduced the spontaneous running wheel activity of mice and maintained this reduction for up to 8 weeks [31]. Acute stress significantly reduced the movement distance and the central residence time of mice [32]. Our results also showed that the movement distance of mice decreased significantly with the extension of cold exposure at various temperatures. The main reason for this reduction may be the decrease in limb flexibility and the imbalance of energy homeostasis caused by a cold environment. However, the initial response of our recipient mice increased, and we observed a rapid and complete recovery of this change, which may be caused by the successful response of mice to cold stress. This has because some suitable and mild and limited cold stimulation is beneficial to the body [33]. The spontaneous movement results of mice exposed to cold at 22 °C proved this view. The vertical rearing activity and the horizontal central activity frequency reflect its exploration behavior in the OFT. Exploratory rearing is sensitive to the environment/acute stress and is reduced under more adverse conditions [7]. The rearing times of vertical activities is more indicative of adaptation and satisfaction with the environment than exploratory behavior [34]. Therefore, the central residence time of mice was used to evaluate their exploratory behavior in our study. Our results showed that the exploratory behavior of mice under cold exposure was significantly reduced, except at the beginning of 22 °C cold exposure. Noise stress and restraint stress significantly impaired horizontal and vertical the exploratory behavior of mice [35]. However, there was an abnormal rise in central residence time after 12 h of cold exposure at 16 °C, 10 °C and 4 °C. This may be caused by the increase in foraging behavior caused by feeding restrictions. The feeding behavior and food intake of pigeons increased under chronic cold exposure [22,36]. 

Scientific assessment of animal emotional states is crucial to animal welfare research, although its effective assessment is still challenging [37]. The elevated plus maze test is a valuable tool with high ecological validity and is widely used to measure anxiety-like behavior in rodents [38]. It reflects the anxiety of mice based on the contradiction between the mouse’s natural aversion to open and high open arms and the spontaneous exploration of the novel environment [39]. The mice were exposed to and moved freely across all arms, and residence time and the number of entries in each arm were used to assess anxiety [40]. Although the number of fecal particles reflects the tension of mice, it is affected by biological rhythm, operation and feeding status, resulting in data distortion. Stress triggers anxiety in humans and DA controls anxiety-like behavior [41]. This change is sensitive to environmental changes, especially temperature [42]. For example, heat acclimation changes physiological indexes such as core body temperature, and also leads to the trend of anxiety-like behavior to beneficial [43]. The long-term changes of DA are closely related to the anxiety-like behavior of adult rats under juvenile stress [44]. The closed arms residence time of mice in PMT reflects their avoidance of the novel environment, which assesses their anxiety level [45]. Our results showed a consistent increase in closed arm residence time and serum DA, suggesting that cold exposure increased anxiety-like behavior in mice. However, both have a decrease at the beginning of cold exposure, especially at 22 °C. This may be because the cold exposure intensity at this time is transient and mild. The fluctuation of serum DA under cold exposure at various temperatures is also consistent with this result. 5-HT interacts with DA to affect anxiety and fear-related behaviors [46]. DA and 5-HT are indispensable excitatory neurotransmitters in the central nervous system. The improvement of DA synthesis ability and the release of response to stress are critical characteristics of mental disorders [47]. The increased secretion of central DA under stress has an anxiety effect [48,49], which has been discussed above. Meanwhile, 5-HT is involved in the mechanisms of the stress response, aggressive behavior, anxiety and depression [50,51]. 5-HT affects the secretion of CRH and adrenocorticotropic hormone induced by stress [52]. 5-HT increases vulnerability to negative emotions by regulating behavioral responses to environmental adversity [53]. 

The ability of an organism to deal with the stress response depends on the appropriate participation in its central and peripheral systems [54]. The central HPA axis is activated by stress stimulation to synthesize and release a variety of neuroendocrine mediators to adapt to the changing environmental needs [55]. HPA axis is the main regulator of nerve and behavior under stress and its dysfunction is related to the increased risk of depression, anxiety, post-traumatic stress disorder and some diseases [56]. Cort reflects the activation level of the HPA axis under stress and the Cort hormone signal is acted on almost all physiological systems to optimize performance according to environmental and physiological needs [55,57]. Cold stress induced excessive Cort in mice and the use of multiple cycles of Cort exposure induced anxiety-like behavior [4,58,59]. Chronic repeated injection of corticosterone induced changes in sleep patterns and decreased foraging behaviors in mice and increased negative maladaptive behaviors such as reward-seeking and effort-related behaviors in mice [58,60,61,62]. Meanwhile, changes in circulating Cort also affected motor motivation and physical ability of motor behavior in mice, such as increased daily wheel running distance, duration and speed and more closed arms residence time of PMT and decreased home-cage activity [63]. Our results showed that the fluctuation of serum Cort was highly consistent with the changes in spontaneous movement and anxiety-like behavior in mice. CRH controls the HPA axis and is a critical regulator of various behaviors and stress responses and endocrine [64]. Localized injection of CRH into the medial prefrontal cortex significantly increased anxiety-related behavior in the PMT [65]. CRH and Cort are hyperactive in the peripheral blood of patients with depression [66]. The LC/NE is activated by stressors to secrete epinephrine and norepinephrine and coordinate endocrine, behavioral and other physiological responses [67]. NE affects individual behavior, including the regulation of alertness, arousal, attention, motivation, reward, learning and memory [68]. The strong activation of the E pathway promotes that unpleasant emotions is "covered" by strong stress to obtain better emotional tolerance in the emotion-centered stress coping model [69]. This may be one of the main reasons for the improvement of anxiety-like behavior in the initial stage of PMT. This will also increase risk-seeking behavior, leading to changes in exploration behavior. Our results suggest a potential link between these two hormones and emotion and behavior. 

## 5. Conclusions

To summarize, mild acute cold exposure promoted the spontaneous movement, increased exploratory behavior and improved anxiety. As the intensity of cold exposure increases, cold exposure had a negative effect on spontaneous movement, exploratory behavior and emotion. The physiological basis of these behavioral and emotional changes in mice under different intensity cold stimulation is the fluctuation of Cort, CRH, E, NE, DA and 5-HT.

## Data Availability

The datasets used during the current study are available from the corresponding authors on reasonable request.
